# Biological Response of the Peri-Implant Mucosa to Different Definitive Implant Rehabilitation Materials

**DOI:** 10.3390/polym16111534

**Published:** 2024-05-29

**Authors:** María Baus-Domínguez, Elena Oliva-Ferrusola, Serafín Maza-Solano, Gonzalo Ruiz-de-León, María-Ángeles Serrera-Figallo, José-Luis Gutiérrez-Perez, Daniel Torres-Lagares, Laura Macías-García

**Affiliations:** 1 Department of Stomatology, Faculty of Dentistry, University of Seville, C/Avicena S/N, 41009 Seville, Spain; elenaof2290@gmail.com (E.O.-F.); serafinmazasolano@gmail.com (S.M.-S.); ruizdeleong@gmail.com (G.R.-d.-L.); maserrera@us.es (M.-Á.S.-F.); jlgp@us.es (J.-L.G.-P.); danieltl@us.es (D.T.-L.); 2 Department of Cytology and Normal and Pathological Histology, School of Medicine, University of Seville, Av. Sánchez Pizjuán S/N, 41009 Seville, Spain

**Keywords:** histological evaluation, dental materials, gingival fibroblast, mucointegration, dental implants, zirconium, polymethylmethacrylate, lithium disilicate

## Abstract

Background: Sealing the peri-implant tissue is a determining factor for long-term implant survival. In the transmucosal region, the cervical fraction of the prosthetic crown is in contact with these tissues, so mucointegration will also be influenced by the biomaterial used for the prosthetic restoration. This study aims to compare the tissue response generated by definitive restorative materials and polymeric materials from a histological point of view. Methods: This study performed an observational prospective cohort study in which biopsies of the peri-implant mucosa were taken after placement of implant-supported prosthetic restorations made of different materials (zirconium oxide, lithium disilicate, and PMMA). Results: A statistically significant difference was observed in the increase in the thickness of the non-keratinized epithelium when comparing the definitive materials (zirconium oxide/lithium disilicate) vs. the provisional material (PMMA) and in the number of collagen fibers when comparing zirconium oxide and lithium disilicate. Conclusions: This study found that zirconia is the material that presents the most adequate biological response of peri-implant tissues. It shows a lower intensity of inflammatory cellular content, a total normality in the number of collagen fibers (the arrangement of the fibers is normal in 90% of the cases), and vascular proliferation of connective tissue in 83% of the cases. These parameters make it a material with a predictable response. Similarly, only the following slight statistically significant differences between the definitive and provisional materials are observed, indicating that the biological response generated by the provisional material (PMMA) is not very different from that obtained with the placement of the definitive restoration.

## 1. Introduction

Since Brannemark’s fortuitous discovery of osseointegration in 1952, and from the 1970s onwards, significant efforts have been invested in developing dental implants, which have become an indispensable part of today’s dentistry [1]. Long-term global studies show that the survival rate of implants is 96.1% (95% CI: 87.3, 98.9%) at five years [1,2,3,4].

Dental implants comprise a system in which an alloplastic device surgically inserted in the alveolar ridge is connected through an abutment to a superstructure (implant-supported prosthesis) that replaces the missing tooth [5]. Part of the successful survival of dental implants will depend on the transmucosal region, the transition zone separating the osseointegrated implant in the bone, and the prosthetic tooth exposed to the oral cavity. This transmucosal region contains gingival fluids, saliva, and bacteria and mainly comprises epithelium and connective tissue [6].

The sealing of the peri-implant tissue is a determining factor in long-term implant survival [5,7]. The architecture of peri-implant soft tissues is similar to that established around natural teeth [8], composed of a sulcus, junctional epithelium, and with connective tissue insertion. However, their histological features differ in several aspects. The peri-implant epithelium is not similar to the junctional epithelium around the teeth. However, it has some similarities, establishing a hemidesmosomes junction between the epithelial cells and the implant surface. The sulcus is slightly larger, from 1 mm around natural teeth to 3–4 mm in dental implants [9]. The connective tissue shows differences in the arrangement of collagen fibers, which form a dense network oriented parallel to the abutment surface, as well as less cellular and vascular proliferation [8].

In healthy conditions, a mucosal seal is formed around the teeth that do not allow access to aggressive microflora to the underlying bone during tooth eruption. In the case of peri-implant tissues, once they have completed their healing, an effective mucosal seal forms around the transmucosal portion of the implant [10,11]. The hemidesmosomes present at the epithelial junction, immune cells, and the mechanical buffering of the connective tissue ensure a protective mechanical and biological barrier to the underlying bone at this location [8,12]. However, the constant exposure of tissues to microorganisms in the oral cavity makes mucosal healing around transmucosal abutments a significant challenge [10].

The characteristics of peri-implant connective tissue (lower cellularity and vascularity and the parallel arrangement of collagen fibers) could make supracrestal soft tissues more susceptible to peri-implant disease and its progression [8]. In the dental implant region, the ideal healing and regenerative process involves rapid revascularization and effective neoangiogenesis to promote the formation of connective tissue and bone around the implant. The presence of blood vessels is crucial to supply nutrients, oxygen, and immune cells, as well as to facilitate the bone remodeling process. Differences in the arrangement of collagen fibers around implants, with an orientation parallel to the implant abutment and less cellular and vascular proliferation, can negatively affect osseointegration and long-term implant stability. Prevascularized spheroids could improve this environment by providing a scaffold for cell growth and orientation, as well as promoting enhanced neoangiogenesis [13]. For example, if prevascularized spheroids are incorporated into the implant site, they could help establish a microvascular network in the tissue surrounding the implant abutment. This network could enhance the integration and proliferation of connective tissue cells, as well as facilitate the formation of a well-structured collagen network that improves implant support and stability. Furthermore, improved vascularization may lead to a better immune response and accelerate tissue healing and regeneration. Maintenance of the soft tissue peri-implant is essential to preserve long-term oral health and ensure the success of dental implants [8,12,14].

Once osseointegration is consolidated, the main concern is to avoid peri-implant disease: mucositis and peri-implantitis. Bleeding during soft probing (<0.25 N) is the primary indicator to diagnose the presence of mucositis [15]. The histopathologic and clinical conditions that lead to conversion from mucositis to peri-implantitis are not entirely clear, but it is known that mucositis is the precursor to peri-implantitis [15,16]. Peri-implantitis is a pathologic condition of peri-implant tissues, characterized by the inflammation of connective tissue and progressive and irreversible bone loss [16]. The bacterial biofilm adhering to the implant–abutment surfaces and the host response are responsible for triggering the inflammatory response [4]. The disease progresses in a nonlinear and accelerating pattern, leading to the loss of peri-implant bone support [16]. Several studies suggest achieving a better mucosal seal that prevents the passage of bacteria and less bacterial colonization by modifying the biomaterials’ surface to make transepithelial abutments (polished surfaces, drugs, etc.) [6,12,17]. Zirconium (zirconium oxide or zirconia) has gained popularity in dental practice, especially for the fabrication of implant abutments and prostheses due to its good aesthetics and biocompatibility. Zirconia has been documented to have low plaque retention, which is an important contributing factor in preventing bacterial growth and, therefore, may have positive implications in decreasing the risk of infection and peri-implant disease [18,19]. Similarly, dental ceramics are well-known for their excellent aesthetic properties and resistance to wear and corrosion. In terms of antibacterial properties, some ceramics may have a surface that discourages bacterial adhesion, which helps to maintain good health in the tissue surrounding the implant. However, its antibacterial efficacy can vary and is highly dependent on the surface roughness and chemical composition of the material [18]. On the contrary, PMMA does not have inherent antibacterial properties. However, it can be modified by the incorporation of antimicrobial agents, such as metal nanoparticles (e.g., silver or copper) or other antibacterial additives to improve its resistance to bacterial colonization [19,20]. Some have obtained promising results, such as modulating and orienting epithelial tissues to achieve more excellent soft tissue sealing by modifying the surface with technologies that are not yet on the market [6]. However, it should be taken into account that the transmucosal region is not only in contact with the transepithelial abutment but also the cervical fraction of the prosthetic crown, which will be submerged and in contact with these tissues. Thus, the mucointegration of the peri-implant soft tissues will also be influenced by the biomaterial’s surface used for prosthetic restoration.

In recent decades, the number of restorative materials for implant-supported prostheses has increased significantly. Ceramics have gained great popularity due to their many advantages, such as high biocompatibility, more natural appearance, and metal-free structure. Thus, they are characterized by translucency, color stability, low plaque adhesion, wear resistance, and low thermal conductivity [18]. In vitro studies have indicated that they are materials that induce favorable biological responses and exhibit excellent long-term stability [18]. Among ceramic materials, zirconium dioxide has become one of the most widely used restorative materials due to its low cost and high strength [17], and it is known for its high biocompatibility and chemical stability, which contributes to low inflammation and good integration with gingival tissues [18,21]. Zirconia is resistant to corrosion in the moist and acidic environment of the mouth, which helps to maintain its structural stability over time [18]. Similarly, modern materials, such as IPS e.max lithium disilicate from Ivoclar Vivadent, which stands out for its translucent color and good durability, are also available on the market. Both materials can be milled in monobloc using CAD–CAM (computer-aided design–computer-aided manufacturing) technology, which reduces the number of appointments and increases the precision of the final restoration [17].

On the other hand, the materials used for temporary restorations have significantly advanced in their aesthetic and functional characteristics. However, due to their short time in the mouth, their biological properties have been little studied. In a previous study, our team compared the tissue response to materials used as prosthetic temporaries, focusing on histological analysis of the reaction of keratinized tissue, connective tissue, and the inflammatory response to temporary restorations on dental implants [22]. These materials have a long history of use in dentistry but can be susceptible to fracture and crack formation, potentially compromising their stability [20]. They are known to be modified to improve their mechanical properties and resistance to bacterial biofilm growth. However, in their unmodified form, they may not be as stable as ceramic materials or zirconia, especially when subjected to repetitive chewing loads [20]. The stability of these materials also depends on factors such as the design and fabrication of the prosthesis or implant component, patient compliance with care protocols, and precision in fabrication.

It is, therefore, necessary to know whether the biological response generated in the peri-implant soft tissues by provisional materials is similar to that found in the case of the use of definitive restorative materials during the phase of formation and establishment of the surrounding mucosa. On the other hand, the use of monolithic materials seems to present advantages over conventionally veneered ones [23].

This study compares the tissue response generated by definitive prosthetic restorative materials and temporary polymeric materials from a histological point of view. For this purpose, new histological data on connective tissue composition, keratinized epithelium and non-keratinized epithelium, and the inflammatory response to lithium disilicate (IPS™ e.Max CAD LT^®^, IVOCLAR Vivadent, Madrid, Spain), zirconium oxide (Cercon xt ML Multilayer^®^, Dentsply Sirona, Barcelona, Spain), and PMMA (Telio CAD^®^, Ivoclar Vivadent, Madrid, Spain) are examined. This study aimed to determine the influence of these materials on the formation of adhesive structures for sealing the peri-implant mucosa as well as determine which definitive restorative material favors a better tissue response. The null hypothesis of the present study is that the biological response generated in the peri-implant tissues is similar to restorations made of temporary and definitive materials.

## 2. Materials and Methods

### 2.1. Type of Study

The current study is a prospective observational cohort study involving the for-mation of three distinct groups.

This study was approved by the Andalusian Biomedical Research Ethics Coordi-nating Committee (Code US-DTL-2022.1) and complies with all the guidelines of the World Medical Association Declaration of Helsinki: Ethical Principles for Medical Re-search Involving Human Subjects [24]. In compliance with this, all patients received the information sheet and gave informed consent for the intervention in question and par-ticipation in the project.

The patients’ only invasive procedure was the biopsy of the peri-implant tissues of those restorations whose crowns were made of the materials to be studied.

### 2.2. Samples

A group of patients requiring implant-supported prosthetic rehabilitation in the posterior sectors (from premolars to molars) of both arches (maxilla and mandible) were selected.

These were selected after applying the following inclusion and exclusion criteria:

- Inclusion criteria.
  ○ Patients over 18 years of age;
  ○ Patients with posterior partial edentulism requiring implant-supported prosthetic restorations;
  ○ Patients who do not present any absolute contraindication for the placement of dental implants;
  ○ Patients without active periodontal disease.
- Exclusion criteria.
  ○ Patients who are smokers (>10 cigarettes/day);
  ○ Patients with pathology or undergoing treatment that alters bone metabolism or soft tissue healing;
  ○ Patients with poor oral hygiene;
  ○ Patients with uncontrolled periodontal disease;
  ○ Patients with alcoholism or drug problems.

### 2.3. Surgical Protocol for the Placement of Dental Implants

All surgeries were performed by the same oral surgeon without complications. All patients were administered local anesthesia (articaine hydrochloride + epinephrine; Ar-tinibsa^®^ 40 mg/mL + 0.01 mg/mL; Inibsa dental, Barcelona, Spain). Once the area was anesthetized, a mid-crestal incision was made, preserving 2 mm of keratinized tissue on both sides of the incision. A small full-thickness flap was elevated, and the implant bed was prepared with abundant saline irrigation.

The Astra EV system (Astra Tech Implant System^®^ EV, Dentsply Sirona S.A., Barcelona, Spain) is a bone-level dental implant with a defined rough surface, obtained by a patented titanium bombardment technique, followed by a process including treatment in dilute hydrofluoric acid. The external part of the collar has micro threads (MicroThread). The type of connection is a conical connection between the implant and the abutment for a firm and stable connection between them (Conical Seal Design).

The diameter of the implants ranged from 3.6 mm to 4.8 mm, depending on the tooth to be replaced and bone availability.

To avoid a second access surgery and take into account the individual conditions of each patient, a healing abutment was screwed in, which had been previously chosen based on the patient’s gingival height.

### 2.4. Description of Restorative Materials

The materials chosen for the fabrication of the prosthetic restorations, according to their properties and purpose, were as follows:

Cercon xt ML Multilayer^®^ (Dentsply Sirona, Barcelona, Spain): an extra translucent multilayer zirconia consisting mainly of zirconium oxide, yttrium oxide 9%, and hafnium oxide <3% (aluminum oxide, silicon oxide, and other oxides <2%). It presents a translucency of 49% (being one of the most translucent zirconia) and a range of colors composed of 16 shades of VITA^®^2 and BL2, achieving a realistic aesthetic. This monobloc presentation allows the use of the CAD–CAM system, facilitating production.

IPS™ e.Max CAD LT^®^ (Ivoclar Vivadent, Madrid, Spain): a glass ceramic formed by a microstructure of lithium disilicate crystals embedded in a glass matrix, used for the fabrication of crowns or as a coating for a metal core. Its characteristics allow a minimally invasive preparation and the use of the CAD–CAM system, achieving adequate adjust-ments. It stands out for its excellent aesthetic properties (desired opalescence, translucency), mechanical properties (high fracture resistance and adaptation), and high technical tolerance. Thanks to the anterior and the assortment of IPS™ e.Max CAD blocks comprising a selection of shades and levels of translucency, great clinical results are achieved. In addition, its durability and enamel-like wear rate make it a very suitable material.

Telio CAD^®^ (Ivoclar Vivadent, Madrid, Spain): blocks of cross-linked polymethyl methyl methacrylate (PMMA) designed for the fabrication of long-term temporary restorations (up to 12 months) using the CAD–CAM system. Its industrial polymerization process makes it highly homogeneous, avoiding polymerization shrinkage. Its physical properties include resistance to bending and fracture (130 ± 10 MPa), almost total transparency, low water absorption, and low density. Its aesthetic properties include its range of colors and lasting stability. At the same time, its advantages, such as ease of repair and good marginal adaptation, should be valued. Its finishing and fitting can be performed with conventional tungsten carbide cross-cutting burs.

### 2.5. Design and Fabrication of Restorations

Once the three months of osseointegration had elapsed, digital measurements were taken using the Primescan™ intraoral scanner (Software Connect 5.1, Dentsply Sirona S.A., Barcelona, Spain) for the design on Atlantis titanium nitride abutments (Dentsply Sirona S.A., Barcelona, Spain).

Once the abutment design was completed, the prosthetic crowns were fabricated. The restorations were fabricated from zirconia (Cercon xt ML^®^), lithium disilicate (IPS™ e.Max CAD LT^®^), or PMMA (Telio CAD^®^). They were aesthetically and functionally adapted to the abutment design, the antagonist arch, and the adjacent teeth.

The design of the restorations was extended more than 2 mm subgingivally to ensure the contact of the restorative material and the peri-implant mucosa. To obtain as smooth a surface as possible and thus avoid bacterial adhesion, all restorations were polished with a silicone disc (Edenta Exa Cerapol UM, EDENTA AG, Hauptstrasse 7, CH-9434 AU/SG, Switzerland), a mounted goat hair disc brush, and finally, a felt disc.

The crowns were cemented onto the Atlantis abutments out of the mouth with Relyx dual-type resin cement (Relyx™ Unicem 2 Automix Self-Adhesive Resin Cement, 3M Oral Care, 2510 Conway Avenue, St. Paul, MN 55144-1000 USA) for the definitive restorative materials and with zinc oxide–eugenol for the temporary material.

Subsequently, all the restorations were screwed at a torque force of 25 Ncm^2^, according to the manufacturer’s recommendation.

### 2.6. Biopsy

All biopsies were taken between 21 January 2021 and 28 July 2021, with an average of 180 days from the placement of the restoration of the material to be studied. The protocol was performed under local anesthesia (lidocaine 2% and epinephrine 1:100,000, Xilonibsa, Inibsa, Barcelona, Spain). Once the area was anesthetized, the sample was taken through an external bevel incision up to the vestibular epithelium in contact with the material, measuring 1–2 × 4–5 mm. Finally, the sample was preserved in a sterile jar with 40% formalin and sample coding for single-blind analysis.

Each sample was processed in 4 mm sections fixed in formalin for embedding in kerosene. Subsequently, staining was performed with hematoxylin–eosin and Masson’s trichrome. The former allowed the identification of changes in the vascular and cellular components of the epithelium and connective tissue, while the latter showed the collagen fibers.

All histological sections were digitally archived by taking photographs (Color Camera Nikon DS-Fi3, v. 100.06.3307.E9) under the optical microscope at different magnifications OLYMPUS VANOX AHBT3 (Olympus Corp., Tokyo, Japan)with 4×, 10×, 20×, and 40× objective lenses.

Subsequent image processing was performed with NIS Elements Imaging Software (v.5.21.00).

After the biopsies had been taken, those patients who had been fitted with a tem-porary PMMA restoration (Telio CAD^®^) had a definitive restoration made of zirconium oxide (IPS e.max ZirCAD™ Multi, IVOCLAR Vivadent, Madrid, Spain) cemented onto the previously screw-retained Atlantis abutment.

Finally, to ensure proper surgical site healing, all patients were revisited one month after the biopsy.

### 2.7. Statistical Analysis

Four dichotomous crosses were performed: zirconium/disilicate (together) vs. PMMA, zirconium vs. PMMA, disilicate vs. PMMA, and zirconium vs. disilicate.

#### 2.7.1. Descriptive Analysis

A complete descriptive analysis of the following variables was performed:

- Qualitative:
  ○ Primary location of inflammatory activity in the epithelium;
  ○ Lateral cellular composition of the inflammatory activity;
  ○ Cellular composition of the inflammatory activity;
  ○ Number of connective tissue collagen fibers;
  ○ Arrangement of the collagen fibers of the connective tissue;
  ○ Vascular proliferation of connective tissue.
- Quantitative:
  ○ Increased thickness of keratinized epithelium;
  ○ Size of the ridges in the keratinized epithelium (mm);
  ○ Exocytosis. Number of lymphocytes per mm^2^ in the keratinized epithelium;
  ○ Exocytosis. Number of nuclear polymorphs per mm^2^ in the keratinized epithelium;
  ○ Exocytosis. Total number of cells per mm^2^ in the keratinized epithelium;
  ○ Parakeratosis. Exocytosis. Number of nuclei per mm^2^ in the keratinized epithelium;
  ○ Increased thickness of the non-keratinized epithelium (mm);
  ○ Size of the ridges in the non-keratinized epithelium;
  ○ Intensity of inflammatory activity. Percentage of inflammatory cells;
  ○ Number of connective tissue papillae.

#### 2.7.2. Normality of Numerical Variables

The Kolmogorov–Smirnov test was applied to determine normality.

#### 2.7.3. Cross-Tabulation of the Material and Qualitative Variables

The Chi^2^ test was carried out.

To determine the groups that make the difference, we used Haberman’s corrected typified residuals, which allowed us to obtain the significance of the cells independently; this significance implies that the percentage of the cell is statistically different from that corresponding to the total sample.

#### 2.7.4. Cross-Tabulation of the Material and Numerical Variables

A one-way ANOVA was applied for normally distributed variables and the Kruskal–Wallis test for the rest.

## 3. Results

### 3.1. Patients and Study Groups

A total of 33 patients were candidates for the study. The study groups were as follows according to the restorative material:

- IPS™ e.Max CAD LT^®^: nine patients;
- Cercon xt ML Multilayer^®^: 12 patients;
- Telio CAD^®^: 12 patients.

### 3.2. Histological Analysis

**Figure 1:**
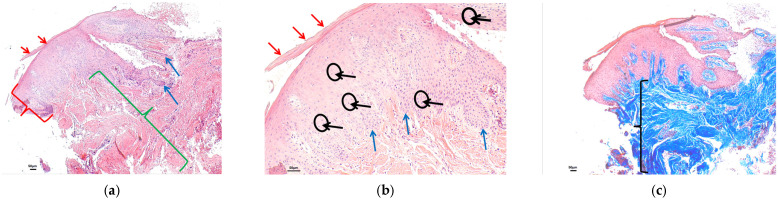
Histological section with hematoxylin–eosin and Masson’s trichrome stain of control gingiva from a healthy patient: (**a**) HE (4×): stratified squamous epithelium (red key) with hyperkeratosis (red arrows) without alterations in normality. Papillary projections that extend into the connective tissue (blue arrows). Compact connective tissue (green key) without alterations to normality; (**b**) HE (10×): Figure 1a at higher magnification, showing a stratified squamous epithelium with hyperkeratosis (red arrow). The epithelium consists of well-structured keratinocytes (black arrows and circles) that form papillae that extend into the submucosal tissue (blue arrows). No inflammatory cells are identified; (**c**) Masson’s trichrome (10×): submucosal tissue consisting of collagen bundles, identified by blue staining, without alterations in their structure, maintaining a parallel arrangement between them (black key).

For a better understanding of the histological analysis, a biopsy of a control gingiva is taken from a healthy patient (Figure 1). Normal gingiva has a stratified squamous epithelium. This epithelium usually shows thickening of the stratum corneum, known as hyperkeratosis. Keratin abnormalities associated with hyperkeratosis usually involve loss of the nucleus in the upper strata (orthokeratotic hyperkeratosis). However, in the mucosa, it is normal to find areas of keratinization with preserved nuclei in the stratum corneum, known as parakeratotic hyperkeratosis or parakeratosis (Figure 1a—epithelium). In the same image, at higher magnification, we can see in detail the cells that make up the epithelium, which are mainly keratinocytes, as well as other isolated cells in the stratum basale, such as melanocytes. Cells of an inflammatory nature are usually not identified. The gingival epithelium usually has epithelial extensions that extend into the connective tissue (Figure 1b). Connective tissue consists of a compact, well-organized network of collagen fibers arranged parallel to each other. Between the bundles of collagen fibers, some cells, such as fibroblasts, and very few cells of an inflammatory nature, such as lymphocytes, can be identified. We observed little representation of vascular proliferation in the upper layers of connective tissue (Figure 1a—submucosal tissue). The analysis of the collagen fibers of the connective tissue is performed by evaluating the result of the special technique known as Masson’s trichrome, which represents the method of collagen staining, during which aniline blue dye binds to the collagen causing it to turn distinctly blue. It is also used to visualize alterations in the collagen bundle network, such as increased collagen accumulation, associated with possible scar tissue, or decrease or alteration in its arrangement and architecture (Figure 1c).

#### 3.2.1. IPS™ e.Max CAD LT^®^

**Figure 2:**
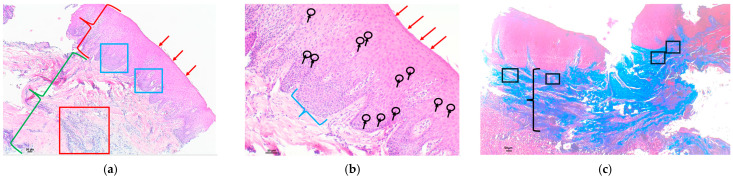
Histological section with hematoxylin–eosin and Masson’s trichrome stain of the peri-implant mucosa around an IPS™ e.Max CAD lithium disilicate restoration: (**a**) HE (4×): stratified squamous epithelium (red key) with parakeratosis (red arrows), as well as thickening and fusion of papillae projected into connective tissue (blue squares). Submucosal tissue (green key) with a lymphocytic inflammatory infiltrate, represented as an extension of intense basophilic cellularity with punctate morphology, moderately localized in the intermediate zone (red square); (**b**) HE (10×): Figure 2a at higher magnification, with a stratified squamous epithelium with parakeratosis (red arrows), where a fusion of papillae formed by well-organized keratinocytes can be seen (blue key). Mild intercellular lymphocytic infiltration at the intraepithelial level (black arrows and circles); (**c**) Masson’s trichrome (4×): submucosal space with increased proliferation of connective tissue bundles identified by blue staining, without alterations in their arrangement (black key). No increase in vascular proliferation (black squares).

**Figure 3:**
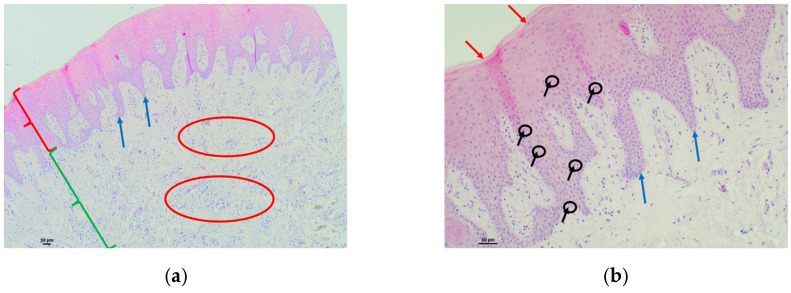

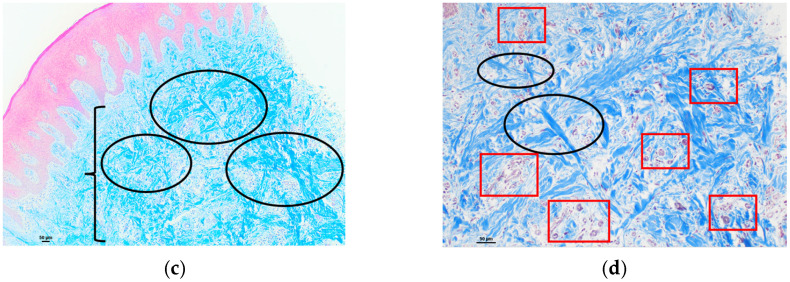
Histological section with hematoxylin–eosin and Masson’s trichrome stain of the peri-implant mucosa around an IPS™ e.Max CAD lithium disilicate restoration: (**a**) HE (4×): stratified squamous epithelium (red key) showing the preservation of thin stacks of well-organized keratinocytes projecting toward the connective tissue (blue arrows). Submucosal tissue (green key) with a lymphocytic inflammatory infiltrate, represented as an extension of intense basophilic cellularity of punctiform morphology, of mild character located in the intermediate zone (red ellipses); (**b**) HE (10×): (**a**) at higher magnification, with a stratified squamous epithelium with parakeratosis foci (red arrows) and thin piles (blue arrows). Intraepithelial polymorphonuclear infiltration at the intercellular level is very mild (black arrows and black circles); (**c**) Masson’s trichrome (4×): submucosal space with increased proliferation of connective tissue bundles, identified by blue staining (black key). Abnormal arrangement of the connective tissue with loss of the parallel arrangement of the collagen bundles, with the presence of areas of collagen fibers with disordered and anarchic distribution (black ellipses); (**d**) Masson’s trichrome (10×): abnormal arrangement of the connective tissue where collagen bundles are identified with a disordered and anarchic distribution with loss of the parallel arrangement (black–black ellipses). Increased vascular proliferation between the disordered collagen bundles (red squares).

Examples of analyzed histological sections of peri-implant mucosal tissue around lithium disilicate restorations are shown below (Figure 2 and Figure 3).

In the second figure, the mucosal tissue in the area adjacent to the implant where the lithium disilicate material was used showed a stratified squamous epithelium with foci of keratinization with preserved nuclei, a phenomenon known as parakeratosis, as well as thickening and fusion of papillae projecting toward the connective tissue (Figure 2a—epithelium). In the submucosal tissue, a lymphocytic inflammatory infiltrate was detected, represented as an intense basophilic cellular component with punctate morphology, located in the intermediate zone. This lymphocytic infiltrate was higher than the percentage considered normal and was therefore assessed as moderate (Figure 2a—submucosal tissue). In the same image, at higher magnification, mild infiltration of intercellular lymphocytes was identified at the intraepithelial level, between the keratinocytes that make up the mucosal epithelium, a phenomenon known as exocytosis (Figure 2b). The Masson’s trichrome technique, which highlights the collagen fibers by means of blue staining, allowed us to identify a submucosal space with an increased concentration and proliferation of collagen bundles, but these remained parallel to each other, without identifying any alterations in their structure. A slight proliferation of vessels was identified in the upper area of the connective tissue, without alterations to normality (Figure 2c).

In the third figure, the mucosal tissue in the area adjacent to the implant where the lithium disilicate material was used showed a stratified squamous epithelium with orthokeratotic hyperkeratosis and very isolated foci of keratinization with conserved nuclei, a phenomenon known as parakeratosis. The preservation of thin papillae consisting of well-organized keratinocytes, which project into the connective tissue, is identified (Figure 3a—epithelium). In the submucosal tissue, a lymphocytic inflammatory infiltrate was detected, represented as an intensely basophilic cellular component with a punctate morphology, located in the intermediate zone. This lymphocytic infiltrate was slightly higher than the percentage considered normal and was therefore evaluated as mild (Figure 3a—submucosal tissue). In the same image, at higher magnification, very mild polymorphonuclear infiltration was detected at the intraepithelial level between the keratinocytes that make up the epithelium, a finding known as exocytosis (Figure 3b). Masson’s trichrome technique, which shows the collagen fibers by means of blue staining, allowed us to identify a submucosal space with an increase in the concentration and proliferation of collagen bundles (Figure 3c), as well as to detect an alteration in their arrangement, with the loss of the parallel arrangement between the collagen fibers, allowing us to detect perpendicular crosslinking between them, giving rise to a disordered and anarchic distribution, which suggests a possible lesion or scar zone. A proliferation of vessels in the upper area of the connective tissue was identified in excess of that considered normal, with a proliferation of new small vessels between the disordered collagen bundles (Figure 3d).

#### 3.2.2. Telio CAD^®^

**Figure 4:**
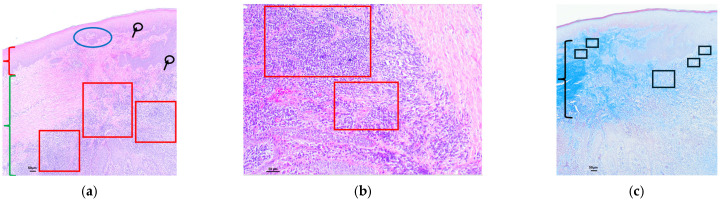
Histological section with hematoxylin–eosin and Masson’s trichrome stain of the peri-implant mucosa around a Telio CAD^®^ (PMMA) restoration: (**a**) HE (4×): stratified squamous epithelium (red key) with parakeratotic hyperkeratosis, as well as thickening, shortening, and areas of fusion of papillae projecting toward the connective tissue (blue ellipse). Intraepithelial infiltration of polymorphonuclears is identified at the intercellular level with a very mild character (black arrows and circles). Connective tissue (green key) with a lymphocytic inflammatory infiltrate, represented as an extension of intensely basophilic punctiform morphology cellularity, of severe character located in the intermediate zone with deep extension (red squares); (**b**) HE (10×): (**a**) at higher magnification, with connective tissue with a severe lymphocytic inflammatory infiltrate, located in a deep area represented as an extension of intensely basophilic cellularity of punctiform morphology that is continuously arranged (red squares); (**c**) Masson’s trichrome (4×): submucosal space with a slight increase in proliferation of connective tissue bundles identified by blue staining, without alterations in their arrangement (black key). No increase in vascular proliferation (black squares).

**Figure 5:**
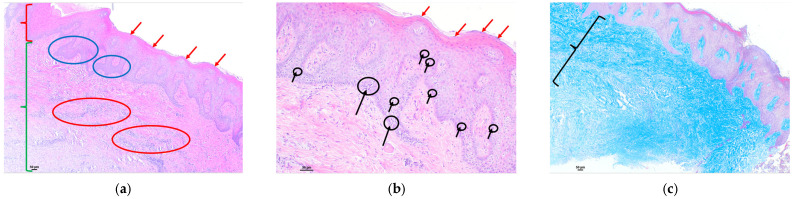
Histological section with hematoxylin–eosin and Masson’s trichrome stain of the peri-implant mucosa around a Telio CAD^®^ (PMMA) restoration: (**a**) HE (4×): stratified squamous epithelium (red key) with parakeratotic hyperkeratosis (red arrows), as well as thickening and fusion of papillae projecting into the connective tissue (blue ellipse). Connective tissue (green key) with a moderate lymphocytic inflammatory infiltrate, represented as an extension of intensely basophilic cellularity with punctate morphology, located in the intermediate zone (red ellipses); (**b**) HE (10×): (**a**) at higher magnification, with a stratified squamous epithelium with parakeratotic hyperkeratosis (red arrows). Intraepithelial lymphocytes are present between the moderate cellular spines (black arrows and circles); (**c**) Masson’s trichrome (4×): submucosal space with increased proliferation of connective tissue bundles, identified by blue staining, without alterations in their structure, maintaining the parallel arrangement between them (black key).

Examples of analyzed histological sections of peri-implant mucosal tissue around PMMA restorations are shown below (Figure 4 and Figure 5).

In the fourth figure, the mucosal tissue in the area adjacent to the implant where the PMMA material was used showed a stratified squamous epithelium, with areas of keratinization with preserved nuclei, a phenomenon known as parakeratosis, as well as thickening, shortening, and areas of fusion of papillae projecting into the connective tissue. Very mild intercellular polymorphonuclear infiltration was also detected at the intraepithelial level, between the keratinocytes that constitute the mucosal epithelium (exocytosis) (Figure 4a—epithelium). In the same image, we focused our attention on the submucosal tissue, where a significant lymphocytic inflammatory infiltrate was detected, represented as an extension of intensely basophilic cellularity with a punctiform morphology that is arranged continuously in an intermediate zone, extending in depth. This lymphocytic infiltrate was much higher than the percentage considered normal and was therefore assessed as severe (Figure 4a—submucosal tissue). At higher magnification, we observed the intense and severe lymphocytic inflammatory infiltrate extending in depth (Figure 4b). Masson’s trichrome technique, which highlights the collagen fibers by means of blue staining, allowed us to identify a submucosal space with an increase in the concentration and proliferation of collagen bundles, but these remained parallel to each other, without identifying any alterations in their structure. A slight proliferation of vessels was identified in the upper area of the connective tissue, which was considered to be normal (Figure 4c).

In the fifth figure, the mucosal tissue in the area adjacent to the implant where the PMMA material was used showed a stratified squamous epithelium, where areas of keratinization with preserved nuclei were identified, a phenomenon known as parakeratosis, as well as the thickening and fusion of papillae projected toward connective tissue (Figure 5a—epithelium). In the submucosal tissue, an inflammatory lymphocytic infiltrate was detected, represented as an extension of intense basophilic cellularity of punctate morphology in the intermediate zone. This lymphocytic infiltrate was higher than the percentage considered normal and was therefore assessed as moderate (Figure 5a—submucosal tissue). In the same image, at higher magnification, moderate infiltration of intercellular lymphocytes was identified at the intraepithelial level, between the keratinocytes that make up the mucosal epithelium (exocytosis) (Figure 5b). The Masson’s trichrome technique, which highlights the collagen fibers by means of blue staining, allowed us to identify a submucosal space with an increased concentration and proliferation of collagen bundles, but these remained parallel to each other, without identifying any alterations in their structure. Little proliferation of vessels was identified in the upper area of the connective tissue, which is considered normal (Figure 5c).

#### 3.2.3. Cercon xt ML Multilayer^®^

**Figure 6:**
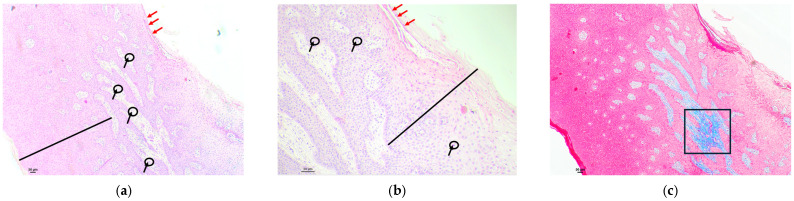
Histological section with hematoxylin–eosin and Masson’s trichrome stain of the peri-implant mucosa around a zirconium oxide restoration (Cercon xt ML Multilayer^®^): (**a**) HE (4×): stratified squamous epithelium with acanthosis (black line) and parakeratotic hyperkeratosis foci (red arrows), as well as thickening and fusion of the papillae projecting toward the connective tissue. Scanty submucosal tissue with a very mild lymphocytic inflammatory infiltrate, represented by very scant basophilic cellularity of punctate morphology, located in the intermediate zone (black arrows and circles); (**b**) HE (10×): (**a**) at higher magnification, with acanthosis (black line) and isolated foci of parakeratotic hyperkeratosis (red arrows). Presence of very isolated intraepithelial lymphocytes between the cell spines (black arrows and black circles); (**c**) Masson’s trichrome (4×): proliferation of bundles of connective tissue identified by blue staining, without alterations in their structure, maintaining the parallel arrangement between them (we identified a higher concentration in the area within the black square).

**Figure 7:**
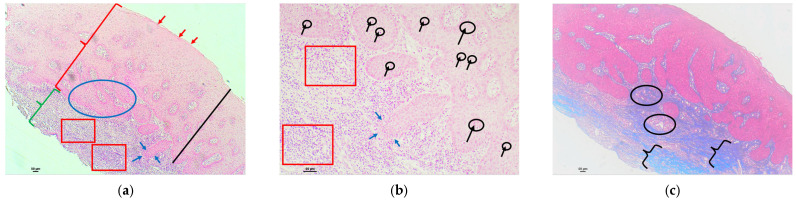
Histological section with hematoxylin–eosin and Masson’s trichrome stain of the peri-implant mucosa around a zirconium oxide restoration (Cercon xt ML Multilayer^®^): (**a**) HE (4×): stratified squamous epithelium (red key) with acanthosis (black line) and parakeratotic hyperkeratosis (red arrows), as well as thickening, shortening, and fusion of the papillae projecting into the connective tissue (blue ellipse). Submucosal tissue (green key) with a severe lymphocytic inflammatory infiltrate represented as an extension of intensely basophilic cellularity of punctate morphology located in the upper and intermediate zone and extending in depth (red squares), showing a slight effacement of the basement membrane and the mucosal–submucosal junction (blue arrows); (**b**) HE (10×): (**a**) at higher magnification, with moderate intraepithelial polymorphonuclear infiltration at the intercellular level (black arrows and circles). Connective tissue with a severe lymphocytic inflammatory infiltrate with mild effacement of the basement membrane and the mucosal–submucosal junction (blue arrows), extending in depth (red squares); (**c**) Masson’s trichrome (4×): proliferation of connective tissue bundles identified by blue co-staining, without alterations in their structure maintaining the parallel arrangement between them (black key). Normal, non-enlarged vascular proliferation (black ellipses).

Examples of analyzed histological sections of peri-implant mucosal tissue around zirconium oxide restorations are shown below (Figure 6 and Figure 7).

In the sixth figure, the mucosal tissue in the area adjacent to the implant where the Zirconia material was used showed a stratified squamous epithelium with an above-normal thickness. This thickening and the thickening of the epithelium affecting all layers of the epithelium is called acanthosis. In addition, areas of keratinization with preserved nuclei were identified, a phenomenon known as parakeratosis, as well as the thickening and fusion of the papillae projecting into the connective tissue (Figure 6a—epithelium). In the submucosal tissue, a very slight lymphocytic inflammatory infiltrate was detected in the intermediate zone (Figure 6a—submucosal tissue). In the same image, at higher magnification, very slight intercellular lymphocyte infiltration was identified at the intraepithelial level, between the keratinocytes that make up the mucosal epithelium (exocytosis) (Figure 6b). The Masson’s trichrome technique, which shows the collagen fibers by means of blue staining, allowed us to identify a submucosal space without alterations in the arrangement of collagen bundles, which remain parallel to each other, without identifying any alterations in their structure. A slight proliferation of vessels was identified in the upper area of the connective tissue, which was considered to be normal (Figure 6c).

In the seventh figure, the mucosal tissue in the area adjacent to the implant where the zirconia material was used showed a stratified squamous epithelium with an above-normal thickness. This thickening and the thickening of the epithelium that affects all layers of the epithelium is called acanthosis. Furthermore, areas of keratinization with preserved nuclei were identified, a phenomenon known as parakeratosis, as well as the thickening, shortening, and fusion of the papillae projected into the connective tissue (Figure 7a—epithelium). In the submucosal tissue, a significant lymphocytic inflammatory infiltrate was detected, represented as an extension of the intense basophilic cellularity of the punctate morphology, located in the upper area, giving rise to points in which the image of the basal lamina is blurred, thus blurring the mucosal–submucosal junction. In the same way, this inflammatory infiltrate was identified in the intermediate zone and was extending in depth. This inflammatory infiltrate was much higher than that considered within the normal range and was therefore considered to be severe (Figure 7a—submucosal tissue). In the same image, at higher magnification, moderate intraepithelial intercellular infiltration of polymorphonuclear cells was identified between keratinocytes that make up the mucosal epithelium (exocytosis), and the slight effacement of the mucosal–submucosal junction can be seen more clearly (Figure 7b). Masson’s trichrome technique, which shows the collagen fibers by means of blue staining, allowed us to identify a submucosal space with no alterations in the proliferation of collagen bundles, nor in their arrangement, as they remained parallel to each other, without identifying any alterations in their structure. A slight proliferation of vessels was identified in the upper area of the connective tissue, which was considered normal (Figure 7c).

### 3.3. Analysis of the Materials Concerning Qualitative and Quantitative Variables

#### 3.3.1. Definitive vs. Provisional Materials (Zirconia/Disilicate vs. PMMA)

The data obtained are summarized in Table 1 and Table 2.

The increase in thickness of the non-keratinized epithelium was substantially more significant in the definitive materials.

#### 3.3.2. Zirconia vs. PMMA

The data obtained are summarized in Table 3 and Table 4.

#### 3.3.3. Disilicate vs. PMMA

The data obtained are summarized in Table 5 and Table 6.

#### 3.3.4. Definitive Materials (Zirconia vs. Disilicate)

The data obtained are summarized in Table 7 and Table 8.

All the collagen fibers were in a normal arrangement in the zirconia-rehabilitated specimens (Table 7).

## 4. Discussion

After implant insertion surgery, cells at the edges of the incision increase and adhere to the implant surface. These can produce basal lamina and hemidesmosomes, creating an epithelial junction similar to the junctional epithelium of natural teeth [8]. Abrahamsson, in 2002 [25], in a study on dogs, showed that, regardless of the type of abutment, a similar epithelial junction was formed, consisting mainly of a thin epithelium and connective tissue rich in fibroblasts in its most apical region. Similarly, other authors have shown in minipigs how zirconium oxide and titanium abutments allow soft tissue integration [26]. However, these studies do not consider the prosthetic restorative material. The most apical region of implant-supported crowns and bridges is subgingival, being in intimate contact with the peri-implant soft tissues. For this reason, the present study aims to compare the biological response generated in the peri-implant soft tissue by materials used for temporary restorations versus materials used in implant-supported definitive restorations and their influence on the improvement of the formation of adhesive structures for sealing the peri-implant mucosa.

Several authors have observed increased collagen fibers in the peri-implant tissue (Ivanovski and Lee, Flores, Deraz). The different causes of this increase may be due to the chemical modification of the abutment surface [8,27] or the compressive loads generated around a loaded implant [24]. However, Flores et al. (2021) [28] demonstrated a significant increase in type V collagen fibers in peri-implant tissues affected by peri-implantitis. Similarly, Deraz et al. (2021) [29] found excessive collagenization of connective tissue in all peri-implantitis cases. Some studies argue that the peri-implant connective tissue, rather than a defense structure, is a chronic inflammatory involvement because it has many collagenase-resistant type V collagen fibers [9]. The present study shows that zirconia presents a normal distribution of collagen fibers in 90–95% of the cases.

The hallmarks of chronic inflammation are continued tissue damage, chronic inflammatory infiltration, and excess fibrous connective tissue (fibrosis). An infiltrate of lymphocytes, macrophages, and plasma cells characterizes the chronic inflammatory reaction. When it is a chronic non-immune provocative inflammatory reaction to a foreign body, it also has a defining feature of activated epithelioid macrophages and multinucleated giant cells. The fibroblasts present usually produce cellular matrix components such as collagen, causing fibrosis (increase in collagen fibers of the connective tissue) and thus resolving the process. When the noxious stimulus is removed, these fibroblasts progressively disappear from the tissue [30].

Different inflammatory cell populations in the junctional epithelium of the peri-implant mucosa result from the microbial challenge posed in the sulcus areas [10]. The lateral cellular composition of the inflammatory activity presents a statistically significant difference in the number of nuclear polymorphs found, increasing in the provisional material concerning the definitive materials. An increase of polymorphonuclear leukocytes (PMN) in peri-implant tissue is considered a significant indicator of inflammation, which could lead to mucositis and peri-implantitis (Berglundh et al., 2018; Schwarz et al., 2018). A single-cell sequencing study supports the involvement of PMNs in inflammation, as it has shown elevated levels of CXCL8, a PMN-attracting cytokine, in peri-implant tissue [31]. Therefore, the presence of PMNs and their association with inflammation could indicate an association with the pathogenesis of peri-implant diseases [32,33].

Similarly, when comparing the response generated by disilicate versus PMMA, a statistically significant difference is found in the increase in exocytosis in the peri-implant tissue around provisional restorations. This increase in exocytosis is another important indicator of inflammation, which, as we have previously mentioned with PMNs, could be related to the possible development of peri-implant diseases [34,35]. The oral mucosa mainly comprises a superficial epithelium attached to the connective tissue by a basal lamina. This oral epithelium is a keratinized or non-keratinized stratified squamous epithelium, whose primary function is defense against mechanical, microbial, and chemical damage [35]. The non-keratinized epithelium has a higher permeability than the keratinized oral mucosa or skin. The healthy gingival epithelium has a mean thickness of 285.04 ± 32.98 µm, its maximum non-pathological value being 333.04 ± 32.98 µm [36]. It is noted that, among the materials examined in this study, no material caused pathological epithelial thickening. It was recorded that zirconium generated a more significant thickening of the non-keratinized epithelium within the physiological mean. We could indicate that an increase in the thickness of the epithelium implies an increase in all cell populations (epithelial cells, Langerhans cells, lymphocytes, etc.) and in the desmosomal junctions and collagen fibers that are established between them, providing a more significant barrier function and defense against microorganisms and pathological substances.

Characteristics such as surface roughness, surface energy, and the relationship of the material with the gingiva influence the state of the peri-implant mucosa and bacterial proliferation [37]. Many current studies present different systems for modifying the zirconium oxide surface to improve its biological properties [38,39,40,41]. A smooth surface promotes good soft tissue sealing and less bacterial colonization [42]. Zirconium oxide is a bioinert ceramic material that allows rapid fibroblastic proliferation on its surface, creating an excellent mucosal barrier. It also inhibits bacterial adhesion due to its hydrophobicity, surface wettability, and reduced surface energy [42]. Monolithic zirconia restorations of nanometer particles have been shown to exhibit improved physical and translucency properties, and their excellent biocompatibility may indicate a high potential for clinical application [38].

Based on the results obtained, we can assume that zirconia is the material presenting the most adequate biological response of peri-implant tissues. It shows a lower intensity of inflammatory cellular content with a predominance of lymphocytes. Likewise, it presents a total normality in the number of collagen fibers. The arrangement of the fibers is normal in 90% of the cases, and vascular proliferation of the connective tissue occurs in 83% of the cases. These parameters make it a material with a predictable response.

## 5. Conclusions

The recent aesthetic advances and the ease of machining, combined with its favorable biological properties, make zirconia the most suitable material for prosthetic restorations, surpassing ceramics thanks to its mechanical properties.

Even so, this study has not found statistically significant differences between definitive and provisional materials, which indicates that the biological response generated by the provisional material (PMMA) is not very different from that obtained with the placement of the definitive restoration.

Despite advances in restorative materials for implant-supported prostheses, there is no scientific evidence on record comparing the mucosal seal obtained after placement of these materials in vivo in humans. Thus, more studies are needed to analyze the behavior of the peri-implant mucosa regarding the different restorative materials, seeking the optimal establishment of a correct transmucosal seal and its maintenance over time.

## Figures and Tables

**Table 1:** Descriptive analysis of the qualitative variables: definitive vs. provisional materials.

Variable	Categories	Zirconia/Disilicate (%)	PMMA (%)	*p*-Value
Primary localization of inflammatory activity in the epithelium	Keratinized epithelium	1 (8.3)	2 (16.7)	
	Intermediate	10 (83.3)	10 (83.3)	
	Non-keratinized epithelium	1 (8.3)	0	
Cellular composition of inflammatory activity	Lymphocytes	15 (83.3)	11 (91.7)	
	PMN	3 (16.7)	1 (8.3)	
Lateral cellular composition of inflammatory activity	No cells	8 (40.0)	1 (8.3)	*p* < 0.05
	Lymphocytes	0	1 (8.3)	
	Lymphocytes, plasma cells	2 (10.0)	0	*p* < 0.05
	Nuclear polymorphs	2 (10.0)	7 (58.3)	
	Nuclear polymorphs, eosinophils	2 (10.0)	0	
	Nuclear polymorphs, plasma cells	6 (30.0)	3 (25.0)	
Number of connective tissue collagen fibers ^1^	Decreased	2 (10.5)	1 (8.3)	
	Normal	15 (78.9)	9 (75.0)	
	Augmented	2 (10.5)	2 (16.7)	
Arrangement of collagen fibers of connective tissue	Normal	18 (94.7)	11 (91.7)	
	Altered	1 (5.3)	1 (8.3)	
Connective tissue vascular proliferation ^2^	Decreased	3 (14.3)	1 (8.3)	
	Normal	15 (71.4)	10 (83.3)	
	Augmented	3 (14.3)	1 (8.3)	

^1^ Decreased: <60%, normal: 60–80%, augmented: >80%. ^2^ Decreased: <35%, normal: 35%, augmented: >35%.

**Table 2:** The quantitative variables’ values (mean ± standard deviation): definitive vs. provisional materials.

Variable	Zirconia/Disilicate	PMMA	*p*-Value
Increased thickness of keratinized epithelium (mm)	0.68 ± 0.37	0.60 ± 0.17	
Size of the ridges in the keratinized epithelium (mm)	6.57 ± 2.69	7.25 ± 2.49	
Exocytosis. Number of lymphocytes per mm^2^ in the keratinized epithelium	17.86 ± 20.67	38.33 ± 37.38	
Exocytosis. Number of nuclear polymorphs per mm^2^ in the keratinized epithelium	22.95 ± 61.31	0	
Exocytosis. Total number of cells per mm^2^ in the keratinized epithelium	40.81 ± 57.67	38.33 ± 37.38	
Parakeratosis. Exocytosis. Number of nuclei per mm^2^ in the keratinized epithelium	33.29 ± 28.59	45.00 ± 56.63	
Increased thickness of non-keratinized epithelium (mm)	0.75 ± 0.49	0.32 ± 0.13	*p* < 0.05
Size of ridges in non-keratinized epithelium	4.50 ± 2.14	4.40 ± 2.55	
Intensity of inflammatory activity. Percentage of inflammatory cells	21.05 ± 21.35	32.71 ± 28.31	
Number of connective tissue papillae	6.05 ± 2.42	6.42 ± 2.07	
Days between placement and sampling	74.81 ± 21.37	70.25 ± 11.69	

**Table 3:** Descriptive analysis of the qualitative variables: zirconia vs. PMMA.

Variable	Categories	Zirconia	PMMA	*p*-Value
Primary localization of inflammatory activity in the epithelium	Keratinized epithelium	1 (8.3%)	2 (16.7%)	
	Intermediate	10 (83.3%)	10 (83.3%)	
	Non-keratinized epithelium	1 (8.3%)	0	
Cellular composition of inflammatory activity	Lymphocytes	9 (90.0%)	11 (91.7%)	
	PMN	1 (10.0%)	1 (8.3%)	
Lateral cellular composition of inflammatory activity	No cells	3 (27.3%)	1 (8.3%)	
	Lymphocytes	0	1 (8.3%)	
	Lymphocytes, plasma cells	1 (9.1%)	0	*p* < 0.05
	Nuclear polymorphs	1 (9.1%)	7 (58.3%)	
	Nuclear polymorphs, eosinophils	2 (18.2%)	0	
	Nuclear polymorphs, plasma cells	4 (36.4%)	3 (25.0%)	
Number of connective tissue collagen fibers ^1^	Decreased	0	1 (8.3%)	
	Normal	15 (78.9%)	9 (75.0%)	
	Augmented	0	2 (16.7%)	
Arrangement of collagen fibers of connective tissue	Normal	10 (90.9%)	11 (91.7%)	
	Altered	1 (9.1%)	1 (8.3%)	
Connective tissue vascular proliferation ^2^	Decreased	1 (8.3%)	1 (8.3%)	
	Normal	10 (83.3%)	10 (83.3%)	
	Augmented	1 (8.3%)	1 (8.3%)	

^1^ Decreased: <60%, normal: 60–80%, augmented: >80%. ^2^ Decreased: <35%, normal: 35%, augmented: >35%.

**Table 4:** The quantitative variables’ values (mean ± standard deviation): zirconia vs. PMMA.

Variable	Zirconia	PMMA	*p*-Value
Increased thickness of keratinized epithelium (mm)	0.73 ± 0.39	0.60 ± 0.17	
Size of the ridges in the keratinized epithelium (mm)	5.75 ± 3.11	7.25 ± 2.49	
Exocytosis. Number of lymphocytes per mm^2^ in the keratinized epithelium	23.92 ± 23.19	38.33 ± 37.38	
Exocytosis. Number of nuclear polymorphs per mm^2^ in the keratinized epithelium	19.75 ± 56.25	0	
Exocytosis. Total number of cells per mm^2^ in the keratinized epithelium	43.67 ± 51.68	38.33 ± 37.38	
Parakeratosis. Exocytosis. Number of nuclei per mm^2^ in the keratinized epithelium	33.08 ± 29.81	45.00 ± 56.63	
Increased thickness of non-keratinized epithelium (mm)	0.73 ± 0.48	0.32 ± 0.13	*p* < 0.05
Size of ridges in non-keratinized epithelium	4.00 ± 2.73	4.40 ± 2.55	
Intensity of inflammatory activity. Percentage of inflammatory cells	22.73 ± 21.72	32.71 ± 28.31	
Number of connective tissue papillae	5.33 ± 2.84	6.42 ± 2.07	
Days between placement and sampling	70.58 ± 24.89	70.25 ± 11.69	

**Table 5:** Descriptive analysis of the qualitative variables: disilicate vs. PMMA.

Variable	Categories	Disilicate	PMMA	*p*-Value
Primary localization of inflammatory activity in the epithelium	Keratinized epithelium	0	2 (16.7%)	
	Intermediate	8 (88.9%)	10 (83.3%)	
	Non-keratinized epithelium	1 (11.1%)	0	
Cellular composition of inflammatory activity	Lymphocytes	6 (75.0%)	11 (91.7%)	
	PMN	2 (25.0%)	1 (8.3%)	
Lateral cellular composition of inflammatory activity	No cells	5 (55.6%)	1 (8.3%)	
	Lymphocytes	0	1 (8.3%)	
	Lymphocytes, plasma cells	1 (11.1%)	0	
	Nuclear polymorphs	1 (11.1%)	7 (58.3%)	
	Nuclear polymorphs, eosinophils	0	0	
	Nuclear polymorphs, plasma cells	2 (22.2%)	3 (25.0%)	
Number of connective tissue collagen fibers ^1^	Decreased	2 (25.0%)	1 (8.3%)	
	Normal	4 (50.0%)	9 (75.0%)	
	Augmented	2 (25.0%)	2 (16.7%)	
Arrangement of collagen fibers of connective tissue	Normal	8 (100%)	11 (91.7%)	
	Altered	0	1 (8.3%)	
Connective tissue vascular proliferation ^2^	Decreased	2 (22.2%)	1 (8.3%)	
	Normal	5 (55.6%)	10 (83.3%)	
	Augmented	2 (22.2%)	1 (8.3%)	

^1^ Decreased: <60%, normal: 60–80%, augmented: >80%. ^2^ Decreased: <35%, normal: 35%, augmented: >35%.

**Table 6:** The quantitative variables’ values (mean ± standard deviation): disilicate vs. PMMA.

Variable	Disilicate	PMMA	*p*-Value
Increased thickness of keratinized epithelium (mm)	0.61 ± 0.35	0.60 ± 0.17	
Size of the ridges in the keratinized epithelium (mm)	57.67 ± 1.58	7.25 ± 2.49	
Exocytosis. Number of lymphocytes per mm^2^ in the keratinized epithelium	9.78 ± 14.15	38.33 ± 37.38	*p* < 0.05
Exocytosis. Number of nuclear polymorphs per mm^2^ in the keratinized epithelium	27.22 ± 70.79	0	
Exocytosis. Total number of cells per mm^2^ in the keratinized epithelium	37.00 ± 67.91	38.33 ± 37.38	
Parakeratosis. Exocytosis. Number of nuclei per mm^2^ in the keratinized epithelium	33.56 ± 28.65	45.00 ± 56.63	
Increased thickness of non-keratinized epithelium (mm)	0.78 ± 0.54	0.32 ± 0.13	*p* < 0.05
Size of ridges in non-keratinized epithelium	5.17 ± 0.75	4.40 ± 2.55	
Intensity of inflammatory activity. Percentage of inflammatory cells	18.75 ± 22.08	32.71 ± 28.31	
Number of connective tissue papillae	7.00 ± 1.32	6.42 ± 2.07	
Days between placement and sampling	80.44 ± 15.05	70.25 ± 11.69	*p* < 0.05

**Table 7:** Descriptive analysis of the qualitative variables: Zirconia vs. disilicate.

Variable	Categories	Disilicate	Zirconia	*p*-Value
Primary localization of inflammatory activity in the epithelium	Keratinized epithelium	0	1 (8.3%)	
	Intermediate	8 (88.9%)	10 (83.3%)	
	Non-keratinized epithelium	1 (11.1%)	1 (8.3%)	
Cellular composition of inflammatory activity	Lymphocytes	6 (75.0%)	9 (90.0%)	
	PMN	2 (25.0%)	1 (10.0%)	
Lateral cellular composition of inflammatory activity	No cells	5 (55.6%)	3 (27.3%)	
	Lymphocytes	0	0	
	Lymphocytes, plasma cells	1 (11.1%)	1 (9.1%)	
	Nuclear polymorphs	1 (11.1%)	1 (9.1%)	
	Nuclear polymorphs, eosinophils	0	2 (18.2%)	
	Nuclear polymorphs, plasma cells	2 (22.2%)	4 (36.4%)	
Number of connective tissue collagen fibers ^1^	Decreased	2 (25.0%)	0	
	Normal	4 (50.0%)	11 (100%)	*p* < 0.05
	Augmented	2 (25.0%)	0	
Arrangement of collagen fibers of connective tissue	Normal	8 (100%)	10 (90.9%)	
	Altered	0	1 (9.1%)	
Connective tissue vascular proliferation ^2^	Decreased	2 (22.2%)	1 (8.3%)	
	Normal	5 (55.6%)	10 (83.3%)	
	Augmented	2 (22.2%)	1 (8.3%)	

^1^ Decreased: <60%, normal: 60–80%, augmented: >80%. ^2^ Decreased: <35%, normal: 35%, augmented: >35%.

**Table 8:** The quantitative variables’ values (mean ± standard deviation): disilicate vs. zirconia.

Variable	Disilicate	Zirconia	*p*-Value
Increased thickness of keratinized epithelium (mm)	0.61 ± 0.35	0.73 ± 0.39	
Size of the ridges in the keratinized epithelium (mm)	57.67 ± 1.58	5.75 ± 3.11	
Exocytosis. Number of lymphocytes per mm^2^ in the keratinized epithelium	9.78 ± 14.15	23.92 ± 23.19	
Exocytosis. Number of nuclear polymorphs per mm^2^ in the keratinized epithelium	27.22 ± 70.79	19.75 ± 56.25	
Exocytosis. Total number of cells per mm^2^ in the keratinized epithelium	37.00 ± 67.91	43.67 ± 51.68	
Parakeratosis. Exocytosis. Number of nuclei per mm^2^ in the keratinized epithelium	33.56 ± 28.65	33.08 ± 29.81	
Increased thickness of non-keratinized epithelium (mm)	0.78 ± 0.54	0.73 ± 0.48	
Size of ridges in non-keratinized epithelium	5.17 ± 0.75	4.00 ± 2.73	
Intensity of inflammatory activity. Percentage of inflammatory cells	18.75 ± 22.08	22.73 ± 21.72	
Number of connective tissue papillae	7.00 ± 1.32	5.33 ± 2.84	
Days between placement and sampling	80.44 ± 15.05	70.58 ± 24.89	

## Data Availability

The original contributions presented in the study are included in the article, and further inquiries can be directed to the corresponding authors.

## References

[1] Alghamdi H.S., Jansen J.A. (2020). The development and future of dental implants. Dent. Mater. J..

[2] Howe M.-S., Keys W., Richards D. (2019). Long-term (10-year) dental implant survival: A systematic review and sensitivity meta-analysis. J. Dent..

[3] Schimmel M., Srinivasan M., McKenna G., Müller F. (2018). Effect of advanced age and/or systemic medical conditions on dental implant survival: A systematic review and meta-analysis. Clin. Oral. Implants Res..

[4] van Oirschot B.A.J.A., Zhang Y., Alghamdi H.S., Cordeiro J.M., Nagay B.E., Barao V.A.R., de Avila E.D., van den Beucken J.J.J.P. (2022). Surface Engineering for Dental Implantology: Favoring Tissue Responses Along the Implant. Tissue Eng. Part A.

[5] Kim J.C., Lee M., Yeo I.L. (2022). Three interfaces of the dental implant system and their clinical effects on hard and soft tissues. Mater. Horiz..

[6] Kunrath M.F., Gerhardt M.D.N. (2023). Trans-mucosal platforms for dental implants: Strategies to induce muco-integration and shield peri-implant diseases. Dent. Mater..

[7] Chokaree P., Poovarodom P., Chaijareenont P., Yavirach A., Rungsiyakull P. (2022). Biomaterials and Clinical Applications of Customized Healing Abutment-A Narrative Review. J. Funct. Biomater..

[8] Ivanovski S., Lee R. (2018). Comparison of peri-implant and periodontal marginal soft tissues in health and disease. Periodontology 2000.

[9] Atsuta I., Ayukawa Y., Kondo R., Oshiro W., Matsuura Y., Furuhashi A., Tsukiyama Y., Koyano K. (2016). Soft tissue sealing around dental implants based on histological interpretation. J. Prosthodont. Res..

[10] Tomasi C., Tessarolo F., Caola I., Piccoli F., Wennström J.L., Nollo G., Berglundh T. (2016). Early healing of peri-implant mucosa in man. J. Clin. Periodontol..

[11] Pandoleon P., Bakopoulou A., Papadopoulou L., Koidis P. (2019). Evaluation of the biological behaviour of various dental implant abutment materials on attachment and viability of human gingival fibroblasts. Dent. Mater..

[12] Shim J.S., Kim H.C., Park S.I., Yun H.J., Ryu J.J. (2019). Comparison of Various Implant Provisional Resin Materials for Cytotoxicity and Attachment to Human Gingival Fibroblasts. Int. J. Oral. Maxillofac. Implants.

[13] Lysov A., Saadoun A.P. (2022). Periodontal, Functional, and Esthetic Integration of Peri-Implant Soft Tissue: WHS Concept. J. Oral. Implantol..

[14] Jepsen S., Berglundh T., Genco R., Aass A.M., Demirel K., Derks J., Figuero E., Giovannoli J.L., Goldstein M., Lambert F. (2015). Primary prevention of peri-implantitis: Managing peri-implant mucositis. J. Clin. Periodontol..

[15] Schwarz F., Derks J., Monje A., Wang H.L. (2018). Peri-implantitis. J. Clin. Periodontol..

[16] Kim J.J., Lee J.H., Kim J.C., Lee J.B., Yeo I.L. (2019). Biological Responses to the Transitional Area of Dental Implants: Material- and Structure-Dependent Responses of Peri-Implant Tissue to Abutments. Materials.

[17] Solís Pinargote N.W., Yanushevich O., Krikheli N., Smirnov A., Savilkin S., Grigoriev S.N., Peretyagin P. (2024). Materials and Methods for All-Ceramic Dental Restorations Using Computer-Aided Design (CAD) and Computer-Aided Manufacturing (CAM) Technologies-A Brief Review. Dent. J..

[18] Tetè S., Zizzari V.L., Borelli B., De Colli M., Zara S., Sorrentino R., Scarano A., Gherlone E., Cataldi A., Zarone F. (2014). Proliferation and adhesion capability of human gingival fibroblasts onto zirconia, lithium disilicate and feldspathic veneering ceramic in vitro. Dent. Mater. J..

[19] Singh A.A., Makade C.S., Krupadam R.J. (2021). Graphene nanoplatelets embedded polymer: An efficient endodontic material for root canal therapy. Mater. Sci. Eng. C Mater. Biol. Appl..

[20] Srivastava A., Hazra R., Kumar D., Khattak A., Legha V.S., Kalia D., Verma K. (2022). Graphene: The game changer in dentistry. IP Ann. Prosthodont. Restor. Dent..

[21] Rizo-Gorrita M., Herráez-Galindo C., Torres-Lagares D., Serrera-Figallo M.Á., Gutiérre-Pérez J.L. (2019). Biocompatibility of Polymer and Ceramic CAD/CAM Materials with Human Gingival Fibroblasts (HGFs). Polymers.

[22] Baus-Domínguez M., Maza-Solano S., Vázquez-Pachón C., Flores-Cerero M., Torres-Lagares D., Serrera-Figallo M.Á., Macías-García L. (2023). Behaviour of the Peri-Implant Soft Tissue with Different Rehabilitation Materials on Implants. Polymers.

[23] Sailer I., Strasding M., Valente N.A., Zwahlen M., Liu S., Pjetursson B.E. (2018). A systematic review of the survival and complication rates of zirconia-ceramic and metal-ceramic multiple-unit fixed dental prostheses. Clin. Oral. Implants Res..

[24] General Assembly of the World Medical Association (2014). World Medical Association Declaration of Helsinki: Ethical principles for medical research involving human subjects. J. Am. Coll. Dent..

[25] Abrahamsson I., Zitzmann N.U., Berglundh T., Linder E., Wennerberg A., Lindhe J. (2002). The mucosal attachment to titanium implants with different surface characteristics: An experimental study in dogs. J. Clin. Periodontol..

[26] Bacevic M., Dethier F., Lecloux G., Seidel L., Rompen E., Lambert F. (2023). The Effects of Direct Polymethyl Methacrylate and Zirconia-on-Ti-Base Abutments on Peri-implant Soft Tissue Integration: A Study in Minipigs. Int. J. Prosthodont..

[27] Yamano S., Al-Sowygh Z.H., Gallucci G.O., Wada K., Weber H.P., Sukotjo C. (2011). Early peri-implant tissue reactions on different titanium surface topographies. Clin. Oral. Implants Res..

[28] Flores V., Venegas B., Donoso W., Ulloa C., Chaparro A., Sousa V., Beltrán V. (2021). Collagen quantification in peri-implant soft tissues in human peri-implantitis lesions. Int. J. Morphol..

[29] Deraz E., Soliman O., Saleh R. (2021). Evaluation of Gingival Tissue Pathological Changes in Human Peri-implantitis: Histological, Immunohistochemical and Ultrastructural Study. Egypt. Dent. J..

[30] O’Dowd G., Bell S., Wright S. (2020). Anatomía Patológica Textos, Atlas y Revisión de Hispatología.

[31] Mo J.J., Lai Y.R., Huang Q.R., Li Y.R., Zhang Y.J., Chen R.Y., Qian S.J. (2024). Single-cell sequencing identifies inflammation-promoting fibroblast-neutrophil interaction in peri-implantitis. J. Clin. Periodontol..

[32] Berglundh T., Armitage G., Araujo M.G., Avila-Ortiz G., Blanco J., Camargo P.M., Chen S., Cochran D., Derks J., Figuero E. (2018). Peri-implant diseases and conditions: Consensus report of workgroup 4 of the 2017 World Workshop on the Classification of Periodontal and Peri-Implant Diseases and Conditions. J. Clin. Periodontol..

[33] Noronha Oliveira M., Schunemann W.V.H., Mathew M.T., Henriques B., Magini R.S., Teughels W., Souza J.C.M. (2018). Can degradation products released from dental implants affect peri-implant tissues?. J. Periodontal Res..

[34] Turkoglu O., Efeoglu C., Atmaca H. (2020). Does peri-implant bone loss affect the LL-37 and proteinase 3 levels in peri-implant sulcus fluid?. Int. J. Implant. Dent..

[35] Winning T.A., Townsend G.C. (2000). Oral mucosal embryology and histology. Clin. Dermatol..

[36] Stasio D.D., Lauritano D., Iquebal H., Romano A., Gentile E., Lucchese A. (2019). Measurement of Oral Epithelial Thickness by Optical Coherence Tomography. Diagnostics.

[37] Santillán-Guerra A.M., Ticona-Orellana V.M., Escuza-González S.R., Delgado-Castillo S.M., Huamán-Laredo W.I., Atoche-Socola K.J., Munive-Campos C.A. (2022). Propiedades ópticas y mecánicas del zirconio translúcido como material restaurador óptimo en prótesis fija: Una revisión de la literatura [Optical and mechanical properties of translucent zirconium as an optimal restorative material in fixed prosthesis: A review of the literature]. Rev. Cient. Odontol..

[38] Nasarudin N.A., Razali M., Goh V., Chai W.L., Muchtar A. (2023). Expression of Interleukin-1β and Histological Changes of the Three-Dimensional Oral Mucosal Model in Response to Yttria-Stabilized Nanozirconia. Materials.

[39] Zhang W., Fu W., Wang X., Ye J. (2023). Improving the osseointegration and soft tissue sealing of zirconia ceramics by the incorporation of akermanite via sol infiltration for dental implants. J. Mater. Chem. B.

[40] Huang C., Miao X., Li J., Liang J., Xu J., Wu Z. (2023). Promoted Abutment-Soft Tissue Integration Around Self-Glazed Zirconia Surfaces with Nanotopography Fabricated by Additive 3D Gel Deposition. Int. J. Nanomed..

[41] Han J., Zhang F., Van Meerbeek B., Vleugels J., Braem A., Castagne S. (2021). Laser surface texturing of zirconia-based ceramics for dental applications: A review. Mater. Sci. Eng. C Mater. Biol. Appl..

[42] Sivaraman K., Chopra A., Narayan A.I., Balakrishnan D. (2018). Is zirconia a viable alternative to titanium for oral implant? A critical review. J. Prosthodont. Res..

